# Soluble FMS-Like Tyrosine Kinase: Role in placenta accreta spectrum disorder

**DOI:** 10.12688/f1000research.54719.1

**Published:** 2021-07-21

**Authors:** Sarma Lumbanraja, M Rizki Yaznil, Andre M Siahaan, Bancin Berry Eka Parda

**Affiliations:** 1 Fetomaternal Division, Obstetrics and Gynecology Department, Universitas Sumatera Utara, Medan, Sumatera Utara, 20136, Indonesia; 2 Neurosurgery Department, Universitas Sumatera Utara, Medan, Sumatera Utara, 20136, Indonesia; 3 Resident in Obstetrics and Gynecology Department, Universitas Sumatera Utara, Medan, Sumatera Utara, 20136, Indonesia

**Keywords:** Placenta Accreta, Pregnancy, FIGO, PAS Score, sflt-1

## Abstract

**Background:** Placenta accreta is a pregnancy condition where the placenta's blood vessels attach too deeply to the uterine wall. Incidence of placenta accreta  is increasingly seen today as the rate of cesarean section increases, however, the exact pathophysiology of this condition is still not fully understood. Soluble fms-like tyrosine kinase-1  (sflt-1) as a protein produced by the placenta was found to be decreased in placenta accreta, Therefore we aim  to see if  sflt-1 has a role in the development of placenta accreta. **Methods:** This study involved 40 samples from patients that had been diagnosed with placenta accreta spectrum disorder (case group), and 40 samples from patients with normal pregnancies (control group)  at Rumah Skit Umum Pusat H.Adam Malik (RSUP) Haji Adam Malik Medan, in Indonesia.  Diagnosis of placenta accreta syndrome was based on Placenta Accreta Spectrum  Score (PAS), and International Federation of Gynecology and Obstetrics  (FIGO) classification of placenta accreta spectrum disorder.Analyses  were performed by independent t-test, man Whitney U test, and Kruskal-Wallis analysis test, with a P-value <0.05  considered as statistically significant (95%CI). **Results:** Based on this study, we found that the sFlt-1 level in the case group was lower than the control group. Data analysis using the Kruskal-Wallis test showed that there was a difference in sFlt-1 levels in this study group (p = 0.02), which was further evaluated  with post hoc analysis using Mann. Whitney U test. The results indicated that there were significant differences between the control and PAS 0, PAS1, and PAS 2 (p = 0.043; p = 0.002; p = 0.03). **Conclusion:** sFlt-1 levels decreased in placental invasive pregnancies compared to normal pregnancies, however, this still needs to be investigated further in a multi-center study, considering that sFlt-1 levels are also influenced by ethnicity and other conditions that cannot be excluded in this study.

## Introduction

Placenta accreta is a serious pregnancy condition that can develop when all or part of the placenta adheres to the uterine wall. ^1^ As the cesarean delivery rate increases, it has been noted that placenta accreta cases are on the rise. ^2, 3^ In 2019,at the Haji Adam Malik general hospital, Indonesia, the incidence of this complication had a sharp increase , which resulted in the increased costs of patient care. In 2016 at Adam Malik General Hospital, Medan, Indonesia, placenta accreta mortality rate was very high, as were its complications, such as bleeding, hysterectomy, and pelvic organ injuries. ^2, 4- 9^ Until recently, the diagnosis of placenta accreta spectrum depended on ultrasonography. However, the American College of Obstetricians and Gynecologists (ACOG) and the International Federation of Gynecology and Obstetrics (FIGO) have issued a new terminology for this condition called placenta accreta spectrum, and they have divided the degree of this spectrum according to the ultrasonography, and the depth of invasion at the time of surgery. ^10- 13^ Diagnosis by ultrasonography has many weaknesses as this examination requires expertise and a good standard of ultrasonography equipment. As Indonesia is an archipelagic country, the distribution of doctors with proficient ultrasound expertise in all regions, especially in remote areas, is uneven. In several previous studies, it was reported that several biomarkers can be associated with the placenta accreta spectrum, namely soluble fms-like tyrosine kinase-1 (sFlt-1). As a protein, sFlt-1 is produced by the placenta and it has anti-angiogenetic properties. ^14^ sFlt-1 plays a role in regulating the depth of invasion of the placenta. Previous studies have reported that there was a decrease in sFlt-1 levels in the placenta accreta spectrum. ^15^ Therefore, we aim to investigate the use of sFlt-1 as a diagnostic biomarker for placenta accreta spectrum in the Indonesian population, as an effective replacement for the ultrasound examination.

## Methods

### Population and sample

In this case control study, 80 patients enrolled from February 2020 until June 2020. The patients were divided into either the case group (placenta accreta spectrum), or the control group (normal pregnancy), with 40 patient in each group. Consecutive sampling was preformed for data collection. This research was conducted at the Haji Adam Malik Hospital in Medan, Indonesia, and the patients were selected from the outpatients at the obstetric outpatient polyclinic of this hospital. Patients gave informed consent before participating in this study. Patients in their third trimester of pregnancy who had been diagnosed with a spectrum of placenta accreta, and had planned to give birth by cesarean section, were included in this study. The diagnosis of placenta accreta spectrum was as per ACOG guidelines. ^16^ The examination was performed by two doctor from fetomaternal division who were trained and certified for the use of voluson P6 ultrasound system. Exclusion criteria in this study were patients who were not going to give birth at the Hajj Hospital of Adam Malik, and patients who chose not to participate in this study. Patient characteristics data was obtained from the medical records (see underlying data: https://data.mendeley.com/datasets/y6936wrfty/1). ^17^ Post birth, we assessed the degree of placental invasion of the uterus according to the FIGO criteria previously reported by ACOG, in the case group.

### Ethical approval

The study was conducted after obtaining approval from the research ethics committee of Universitas Sumatera Utara and Haji Adam Malik General Hospital, Medan no: 273/KEP/USU/2020. Written informed consent was obtained from all the participants.

### Data analysis

Before surgery, 3 cc of venous blood from 80 patient was taken and analyzed for serum levels of sFlt-1 in the laboratory of Haji Adam Malik General Hospital in Medan. We used human soluble fimosine like tyrosine kinase -1 ELISA Kit (Bioassay TL; MBS2601616)( see underlying data: https://data.mendeley.com/datasets/y6936wrfty/1). ^17^ Serum levels of sFlt-1 was analysed and compared with the ultrasound-based placenta accreta spectrum disorder classification, and the postoperative FIGO scores of patients on the placenta accreta spectrum.

### Statistical analysis

Analysis in this study was done with the use of SPSS version 26. All data are presented in Mean and standard deviation (SD). Independent Student t-test and Mann Whitney U test were used for the analysis of two variables, while for more than two variables ANOVA and Kruskal Wallis tests were performed. Post Hoc test was used if the ANOVA analysis indicated a significant difference (p < 0.05). The results of the study were considered significant with a p-value < 0.05 (95).

## Result

### Characteristics, proportions, and prevalence of patients

In Table 1, there were no significant differences between the case and control groups except for the number of previous cesarean sections (p < 0.001), history of curettage (p = 0.03) and the distance of previous cesarean section operations (p < 0.001). The comparison of the sFlt-1 levels between the case and control groups indicated that sFlt-1 levels decreased as the degree of placenta accreta spectrum increased. The levels of sFlt-1 based on the placenta accreta spectrum classification compared to controls resulted in a statistically significant difference among the groups (p = 0.004) ( Table 2).

**Table 1.** Patients characteristics.

	PAS (n = 40)	Control (n = 40)	P*
	Mean (SD)	Mean (SD)	
Age (years) Mean+sd	33 (4)	30 (5)	0.08
Bodyweight (kg)	54 (6)	54 (8)	0.72
Height (cm)	145 (20)	150 (5)	0.08
Mid upper arm circumference (cm)	24,8 (3.2)	25,9 (2.5)	0.36
Systolic BP (mmHg)	112 (10)	109 (12)	0.83
Diastolic BP (mmHg)	63 (8)	65 (10)	0.26
Mean arterial pressure	80 (4)	80 (5)	0.49
Gravida	3 (4)	3 (3)	0.09
Parity	2 (2)	2 (2)	0.50
Abortus	0 (0-3)	0 (0-2)	0.13
Gestational age (weeks)	36 (1)	37 (1)	0.68
History of cesarean section	2 (1)	1 (1)	<0.001
History of curretage	0 (0-2)	0 (0-0)	0.03
Last cesarean section (years)	4 (1-7)	1 (0-3)	<0.001

**Table 2.** sflt-1 analysis based on PAS.

	sflt-1 (pg/ml) median (min-max)	*p* *
Control	1246 (824-1636)	0.004
PAS 0	1711 (1367-2055)	
PAS 1	1474 (802-2270)	
PAS 2	1417 (835-2301)	

* *Kruskal Wallis*.

In Table 3, the level of sFlt-1 based on the postoperative FIGO score in the case group was analysed and compared to the control group.Our analysis indicated a difference in sFlt-1 levels, in that obtaining a high FIGO score showed a downward trend in the sFlt-1 level (p = 0.004).

**Table 3.** Post-Hoc analysis of sflt-1 level based on FIGO.

	sflt-1 (pg/ml) median (min-max)	*p* *
Control	1246 (824-1636)	0.002
FIGO 1	-	
FIGO 2	1482 (847-2270)	
FIGO 3	-	
FIGO 4	-	
FIGO 5	1466 (802-2301)	
FIGO 6	1259 (816-2139)	

* *Kruskal Wallis*.

The analysis of the correlation between the sFlt-1 levels and the placenta accreta spectrum classification, based on ultrasonography and FIGO score showed a weak correlation between the groups (r = 0.27, p = 0.015; r = 0.251, p = 0.025) ( Table 4).

**Table 4.** Correlation of PAS and FIGO on sflt-1 serum levels in patients with placental accreta.

Variable	sflt-1 serum (pg/ml) Median (min-max)	*r* *	*p* *
PAS			
PAS 0	1711 (1367-2055)	0.270	0.015
PAS 1	1474 (802-2270)		
PAS 2	1417 (835-2301)		
FIGO			
FIGO 1	1246 (824-1636)	0.251	0.025
FIGO 2	-		
FIGO 3	1482 (847-2270)		
FIGO 4	-		
FIGO 5	-		
FIGO 6	1466 (802-2301)		

* *Spearmen*.

## Discussion

Placenta accreta is a life-threatening condition with an increasing incidence. History of cesarean section is a major risk factor for the placenta accreta spectrum. Diagnosis of this complication is currently by ultrasonography, with several recent studies reporting satisfactory results produced with this mode of diagnosis. ^18^ However, in remote areas where there is a lack of ultrasound equipment and experienced operators, the use of biomarkers can be a solution to this challenge. Recently, studies have reported several proangiogenic and antiangiogenic markers associated with the incidence of placenta accreta spectrum. ^19^ In this condition, there is an imbalance between proangiogenic and antiangiogenic factors, where proangiogenic factors are more dominant and have a significant increase in line with the depth of placental invasion of the uterus. ^15^ The antiangiogenic factor that acts as a regulator is decreased so that the invasion of the placenta is uncontrolled. There are some studies that have established sFlt-1 as proangiogenic and antiangiogenic factors. ^15^ In previous years, sFlt-1 was widely used as a predictor in cases of placenta-related disorders such as preeclampsia. ^20^ This is because in preeclampsia, implantation that occurs is usually superficial due to failure of the transformation of the spiral arteries, therefore there is usually an increase in sFlt-1 levels and decrease in placental growth factor (PLGF) level. sFlt-1 and PLGF as markers were chosen because they are specific to the placenta, although PLGF is also produced by other organs. ^21^ In our study, comparison of the case group with the control group showed no significant difference in the patient characteristics, except for the history of the number of cesarean sections, the duration of the curettage and the length of the previous cesarean section ( Table 1). We also found a decrease in sFlt-1 levels in the placenta accreta spectrum group compared to the control group. Increase in the degree of the spectrum was followed by a decrease in the sFlt-1 level ( Table 2), as well as the FIGO score ( Table 3). Our results additionally showed a weak correlation between sFlt-1 with the accreta spectrum degree and FIGO score. This study is the first study to link sFlt-1 with the degree of placenta accreta spectrum based on ultrasonography and FIGO score. The concentration of sFlt-1 changes throughout the pregnancy, starting with a decrease at 8-12 weeks to 16-20 weeks, with a gradual increase at 26-30 weeks, and a rapid increase at 35-39 weeks. Hirashima et al. reported that the sFlt-1 concentration at 35-39 weeks of gestation was 2000 pg/ml. ^22^ In this study we found lower levels of sFlt-1 (1246 pg/ml) in the control group, and even lower values were observed in the placenta accreta spectrum group (PAS 0, 1711; PAS 1, 1474; PAS 2, 1417). ^22^ These results supports our hypothesis that sFlt-1 is decreased in the placenta accreta spectrum and therefore, it can not regulate placental invasion of the endometrium. Similarly, McMahon et al. had also reported that the lower expression of sFlt-1 in the placenta was associated with placental invasion. ^23^ Additionally, sFlt-1 may play a role in the regulation of cytotropoblast invasion, so that lower sFlt-1 concentration is likely to lead to deeper placental invasion (acreta/increta/percreta). Shainker et al. It has been reported that sFlt-1 expression is reduced in the placental invasion, and non-deep invasion areas of placenta accreta spectrum cases when compared with normal placentas (p = 0.003 and p = 0.001 respectively). Whereas in the placenta from the accreta spectrum cases, the invasive placenta did not show a significant difference in sFlt-1 expression. ^24^ Ostaz et al. reported that there was a decrease in oxidative stress in placenta accreta, and an invasion of the raised placenta and a high oxidative stress in preeclamsia. However, on the placenta accreta spectrum, there was a decrease in the level of oxidative stress, which is probably related to a decrease in the concentration of antio-angiogenic factor, sFlt-1. ^25^ Su et al., reported that aspirin which is usually used as a preeclamptic prophylaxis, has a suppressive effect on sFlt-1. ^26^ Therefore from these findings it can be suggested that suppression of sFlt-1 will increase placental invasion and stimulate placental angiogenesis. Although, whether giving aspirin in normal pregnancy will increase the risk of placenta accreta spectrum incidence has not yet been studied. It can be beneficial to further investigate the effects of aspirin, in order to recognise whether sFlt-1 is a single factor that plays a role in the occurrence of placenta accreta spectrum, or there are other factors involved in placental invasion, such as a history of cesarean section or curettage, which can cause damage to the nitrabuzh layer of the endometrium. This study has several limitations, such as the small sample size . Additionally, we only measured the sFlt-1 levels in the third trimester before the cesarean section was performed. Therefore, we can not report on the effects of sFlt-1 in placenta accreta in the first and second trimesters. Furthermore, we can not rule out the possibility of bias in the assessment of FIGO scores.

## Conclusions

sFlt-1 was found to be decreased in the placenta accreta spectrum, however this result requires further analysis as this correlation was not very strong. Further studies are needed to assess whether sFlt-1 can be used as a biomarker to replace ultrasonography in the diagnosis of the placenta accreta spectrum.

## Data availability

### Underlying data

Mendeley data:Data for Soluble FMS-Like Tyrosine.

Kinase: Role in placenta accrea spectrum disorder https://data.mendeley.com/datasets/y6936wrfty/1. ^17^ This project contains the following underlying data: Data file 1. (sFlt and PLGF ELISA result), Data file 2. (Patient raw data) Data are available under the terms of the Creative Commons Attribution 4.0 International License.

## Consent statement

Written informed consent for publication of the patients’ details was obtained from the patients/a guardian of the patient.

## Author contributions

Sarma N Lumbanraha, role: conceptualization, funding acquisition, resources, supervision, visualization; M Rizki Yaznil, role: investigation, methodology, supervision, visualization; Andre M Siahaan, role: investigation, methodology, supervision, writing – original draft preparation; Berry E P Bancin, role: formal analysis, resources, writing – review and editing.

## Acknowledgements

We would like to thanks the research institute of the University of North Sumatra for funding this research through the TALENTA 2020 program.
