# Supplementary material for: Soluble FMS-Like Tyrosine Kinase-1: Role in placenta accreta spectrum disorder
Source: F1000Res. 2022 Sep 7;10:618. Originally published 2021 Jul 21. [Version 4] doi: 10.12688/f1000research.54719.4 (PMC9478500; doi:10.12688/f1000research.54719.4)
Supplement: Peer review history

# Peer review: Reviewer response for version 4

**DOI:** 10.5256/f1000research.138138.r149779

**Published:** 2022-09-15

**Authors:** Ferdinando Antonio Gulino

**Affiliations:** 1 Department of Obstetrics and Gynecology, Azienda di Rilievo Nazionale e Alta Specializzazione (ARNAS) Garibaldi, Catania, Italy

The authors made all the modifications requested and now it could be accepted for indexing

Is the work clearly and accurately presented and does it cite the current literature?

Yes

If applicable, is the statistical analysis and its interpretation appropriate?

Yes

Are all the source data underlying the results available to ensure full reproducibility?

Partly

Is the study design appropriate and is the work technically sound?

Yes

Are the conclusions drawn adequately supported by the results?

Yes

Are sufficient details of methods and analysis provided to allow replication by others?

Partly

Reviewer Expertise:

Obstetrics

I confirm that I have read this submission and believe that I have an appropriate level of expertise to confirm that it is of an acceptable scientific standard.

# Peer review: Reviewer response for version 4

**DOI:** 10.5256/f1000research.138138.r149778

**Published:** 2022-09-14

**Authors:** Akihide Ohkuchi

**Affiliations:** 1 Department of Obstetrics and Gynecology, Jichi Medical University School of Medicine, Tochigi, Japan

The authors appropriately performed revision. I do not have any more queries

Is the work clearly and accurately presented and does it cite the current literature?

Yes

If applicable, is the statistical analysis and its interpretation appropriate?

Partly

Are all the source data underlying the results available to ensure full reproducibility?

Partly

Is the study design appropriate and is the work technically sound?

Yes

Are the conclusions drawn adequately supported by the results?

Partly

Are sufficient details of methods and analysis provided to allow replication by others?

Partly

Reviewer Expertise:

Obstetrics

I confirm that I have read this submission and believe that I have an appropriate level of expertise to confirm that it is of an acceptable scientific standard.

# Peer review: Reviewer response for version 3

**DOI:** 10.5256/f1000research.77677.r115268

**Published:** 2022-01-04

**Authors:** Ferdinando Antonio Gulino

**Affiliations:** 1 Department of Obstetrics and Gynecology, Azienda di Rilievo Nazionale e Alta Specializzazione (ARNAS) Garibaldi, Catania, Italy

The manuscript "Soluble FMS-Like Tyrosine Kinase-1: Role in placenta accreta spectrum disorder" is original and well structured, gives important novelty to scientific literature but its immediate clinical practice is still limited. The statistical analysis is well conducted and the language is acceptable. It represents an interesting work and it allows focusing the attention on placenta previa when there are some risk factors. The main concern is related to the cesarean section of the patients with PAS disorders: were all planned cesarean sections? how many urgent cesarean sections did you have in your statistics?

 If we consider also the control group we had a mean gestational age at delivery of 37 weeks, is it normal for your population? When was performed the blood sample in the control group considering that we don't know the right time for delivery in a normal pregnancy? A reply by the authors could be interesting. Otherwise, the manuscript is well performed and there are no critical points.

Is the work clearly and accurately presented and does it cite the current literature?

Yes

If applicable, is the statistical analysis and its interpretation appropriate?

Yes

Are all the source data underlying the results available to ensure full reproducibility?

Partly

Is the study design appropriate and is the work technically sound?

Yes

Are the conclusions drawn adequately supported by the results?

Yes

Are sufficient details of methods and analysis provided to allow replication by others?

Partly

Reviewer Expertise:

Obstetrics

I confirm that I have read this submission and believe that I have an appropriate level of expertise to confirm that it is of an acceptable scientific standard, however I have significant reservations, as outlined above.

# Response

**Published:** 2022-03-13

**Authors:** Sarma Lumbanraja

**Affiliations:** North Sumatera University, Indonesia

Thank you for your review

 I would like to answer some question from your analysis

1. In this study, we did not include an emergency case of placenta accreta, because, contraction can change sFlt-1 level in blood, so to minimize bias in our study, case of emergency c-section was ecxluded.
2. In our population and according to our hospital protocol, case of plasenta accreta specttrum disorder will be planned for cesarean section at > 37 weeks gestation according to the cervical length measured by transvaginal ultrasonography. when cervical length < 2,5 cm, and gestational age > 37 weeks, patient planned for cesarean section, but if the cervical length still above 2,5 cm, cesarean section performed at 39 weeks with monitoring.
3.  In the control grup, we do the same protocol as the case group. blood was taken 1 hours before the cesarean section performed. we only limited the case to patient that will performed cesarean section because obstertic indication, for patients with a spontaneous vaginal delivery was excluded from this study, because we cannot predict the time of delivery.

thank you

 best reagards

# Peer review: Reviewer response for version 2

**DOI:** 10.5256/f1000research.76853.r93502

**Published:** 2021-09-13

**Authors:** Akihide Ohkuchi

**Affiliations:** 1 Department of Obstetrics and Gynecology, Jichi Medical University School of Medicine, Tochigi, Japan

Please answer the following queries:

1. Table 1: Is the “Gestational age” at blood sampling or at delivery? You should show both gestational age at blood sampling and at delivery, because the serum levels of sFlt-1 steeply increased even if the difference age is only one week at 36-40 weeks of gestation.
2. Table 4: The values of sFlt-1 in Table 3 and Table 4 are not matched. You should re-check the results.

Is the work clearly and accurately presented and does it cite the current literature?

Yes

If applicable, is the statistical analysis and its interpretation appropriate?

Partly

Are all the source data underlying the results available to ensure full reproducibility?

Partly

Is the study design appropriate and is the work technically sound?

Yes

Are the conclusions drawn adequately supported by the results?

Partly

Are sufficient details of methods and analysis provided to allow replication by others?

Partly

Reviewer Expertise:

Obstetrics

I confirm that I have read this submission and believe that I have an appropriate level of expertise to confirm that it is of an acceptable scientific standard, however I have significant reservations, as outlined above.

# Response

**Published:** 2021-09-13

**Authors:** Sarma Lumbanraja

**Affiliations:** North Sumatera University, Indonesia

thanks for the correction

 1. Gestational age in patient characteristics data is gestational age at the time of blood sampling. Blood samples were taken 1 hour before surgery. enforcement of the PAS score accreta based on ultrasound was carried out 1 day before the cesarean section. Blood collection to assess sFlt-1 levels was carried out 1 hour before the patient underwent a cesarean section and the diagnosis of accre based on FIGO was performed during the duration of the operation.

 2. Sorry for the error in inputting data in this table. we will fix this writing error soon. thanks

 best regards

# Response

**Published:** 2021-11-27

**Authors:** Sarma Lumbanraja

**Affiliations:** North Sumatera University, Indonesia

Hello, 

 I have clarified the question you asked me and I am currently making improvements to my article. Are there any further objections or questions regarding the first question you asked in my research so that I can make further improvements to improve my article? I'm very grateful for your input. 

 Thanks

# Peer review: Reviewer response for version 1

**DOI:** 10.5256/f1000research.58225.r90029

**Published:** 2021-07-22

**Authors:** Akihide Ohkuchi

**Affiliations:** 1 Department of Obstetrics and Gynecology, Jichi Medical University School of Medicine, Tochigi, Japan

General comments:

 The most important matter is the no-adjustment of gestational age at blood sampling. Without adjusting for gestational age, you never conclude that sFlt-1 levels in cases were significantly decreased compared with those in controls.

 Other comments:

1. Title: “Soluble FMS-like tyrosine kinase-1: Role in placenta accreta spectrum disorders” may be better.
2.  Abstract: Line 6: sflt-1 should be sFlt-1. Please reflect this modification in the whole manuscript.
3.  Abstract: Line 6, and Introduction: Line 14: “produced by the placenta” should be changed to “mainly produced by the placenta”.
4.  Abstract: Line 17: “man Whitney” and Line 23: “Mann. Whitney” should be changed to “Mann-Whitney”.
5.  Methods: PAS 0, PAS 1, and PAS 2 should be defined briefly in the Methods. In addition, the FIGO score should be briefly defined in the Methods.
6.  Methods: Statistical analysis: You wrote, “Post Hoc test was used if the ANOVA analysis indicated a significant difference (p < 0.05).” However “Post Hoc test was used if the ANOVA analysis or Kruskal-Wallis tests indicated a significant difference (p < 0.05).” may be a better expression. In addition, you should write how to analyze correlation.
7. Results: Table 1: Is the “Gestational age” at blood sampling or at delivery? You should show both gestational age at blood sampling and at delivery because the serum levels of sFlt-1 steeply increased even if the difference age is only one week at 36-40 weeks of gestation.
8.  Results: Table 1: P-value should be shown till three decimal places, for example, 0.08 should be shown as one of the following numbers: 0.075, 0.076, …, 0.084.
9.  Results: Table 2: Because the levels of sFlt-1 were strongly affected by the gestational age, you should analyze statistics including not only the levels of sFlt-1, but also gestational age (weeks of gestation). One possible statistical method may be generalized linear model which is included in SPSS advanced model. Instead, you can compare sFlt-1 levels after logarithmic transformation using two-way ANOVA.
10.  Table 4: The values of sFlt-1 in Table 3 and Table 4 are not matched. You should re-check the results.
11.  Discussion: Line 28-29: “Shainker *et al.* It has been reported that” Is a grammatical error.

Is the work clearly and accurately presented and does it cite the current literature?

Yes

If applicable, is the statistical analysis and its interpretation appropriate?

Partly

Are all the source data underlying the results available to ensure full reproducibility?

Partly

Is the study design appropriate and is the work technically sound?

Yes

Are the conclusions drawn adequately supported by the results?

Partly

Are sufficient details of methods and analysis provided to allow replication by others?

Partly

Reviewer Expertise:

Obstetrics

I confirm that I have read this submission and believe that I have an appropriate level of expertise to confirm that it is of an acceptable scientific standard, however I have significant reservations, as outlined above.

# Response

**Published:** 2021-08-21

**Authors:** Sarma Lumbanraja

**Affiliations:** North Sumatera University, Indonesia

Thank you for the suggestions and input on my research. me and the team have made some improvements according to the comments you gave on my research. For linear analysis, I tried to include a new chart showing the differences in sFlt-1 levels in the case group and the control group in our patients according to gestational age. for the PAS score assessment, the value we took was 2 days before the patient's cesarean section. For blood collection, it is done 1 hour before cesarean section. I hope this fix can answer all your questions. Thank you for the suggestions and input to improve this article.
